# Does the Motor Level of the Paretic Extremities Affect Balance in Poststroke Subjects?

**DOI:** 10.1155/2014/767859

**Published:** 2014-05-19

**Authors:** Kamal Narayan Arya, Shanta Pandian, C. R. Abhilasha, Ashutosh Verma

**Affiliations:** Pandit Deendayal Upadhyaya Institute for the Physically Handicapped (University of Delhi), Ministry of Social Justice and Empowerment, Government of India, 4 VD Marg, New Delhi 110002, India

## Abstract

*Background*. Poststroke impairment may lead to fall and unsafe functional performance. The underlying mechanism for the balance dysfunction is unclear. *Objective*. To analyze the relation between the motor level of the affected limbs and balance in poststroke subjects. *Method*. A prospective, cross-sectional, and nonexperimental design was conducted in a rehabilitation institute. A convenience sample of 44 patients was assessed for motor level using Brunnstrom recovery stage (BRS) and Fugl-Meyer Assessment: upper (FMA-UE) and lower extremities (FMA-LE). The balance was measured by Berg Balance Scale (BBS), Postural Assessment Scale for Stroke Patients (PASS), and Functional Reach Test (FRT). *Results*. BRS showed moderate correlation with BBS (*ρ* = 0.54 to 0.60; *P* < 0.001), PASS (*r* = 0.48 to 0.64; *P* < 0.001) and FRT (*ρ* = 0.48 to 0.59; *P* < 0.001). FMA-UE also exhibited moderate correlation with BBS (*ρ* = 0.59; *P* < 0.001) and PASS (*ρ* = 0.60; *P* < 0.001). FMA-LE showed fair correlation with BBS (*ρ* = 0.50; *P* = 0.001) and PASS (*ρ* = 0.50; *P* = 0.001). *Conclusion*. Motor control of the affected limbs plays an important role in balance. There is a moderate relation between the motor level of the upper and lower extremities and balance. The findings of the present study may be applied in poststroke rehabilitation.

## 1. Introduction

Balance is an ability to maintain upright position within the base of support during static and dynamic positions [1]. Balance dysfunction, especially during maintenance of erect upright posture and walking, is a common poststroke consequence. Inability to maintain balance reduces functional performance and increases the fall frequency [2]. The dysfunction leads to various musculoskeletal complications multiplying the rehabilitation challenges [3–7].

In stroke, the exact mechanism underlying balance impairment is ambiguous [8]. The factors such as cognition, perception, and biomechanical alterations were found to be responsible for the impairment [9, 10].

A subject with hemiparesis bears more weight on the nonparetic lower extremity leading to asymmetry and impaired erect posture [11]. The weight-bearing asymmetry is associated with the increased postural sway and poor balance [12, 13]. The inability of the nonaffected lower extremity to compensate for the paretic limb also contributes to the postural imbalance [11, 14]. In addition, the arm movements have a considerable role in balance control. The movement of upper limbs usually appears prior to and during loss of balance. By reaching and grasping the outside support, the arms provide a protective function during the fall. The upper limb movements also prevent a fall by shifting the centre of gravity opposite to the direction of imbalance [15, 16]. Due to the arm paresis, poststroke subjects exhibit poor protective function to maintain balance [17]. They demonstrate a deficit in anticipatory and reactive postural adjustments [10]. The impairment of affected lower and upper limbs does not permit the subject to recover from perturbations during functional tasks such as walking [11, 18–20].

In stroke, the voluntary limb movements may have a contribution in balance. However, no study has investigated the relation between the voluntary motor control of the limbs and balance impairment. The objective of the present study was to analyze the relation between the motor level of the affected upper and lower extremities and balance in poststroke subject.

## 2. Methods

### 2.1. Participants

Forty-four patients (34 men and 10 women) attending the Outpatient Occupational Therapy Department of Pandit Deendayal Upadhyaya Institute for the Physically Handicapped were selected for the study. The study protocol was approved by the ethics committee of the institute. The stroke subjects were briefed about the assessment procedure before they signed the informed consent. The subjects who met the following inclusion criteria were selected for the study: (1) chronic stroke (>6 months of onset), (2) ischemic or hemorrhagic stroke, (3) 35 to 65 years of age [21–23], and (4) Functional Ambulation Classification (FAC) [24] level 2 and above. The subjects were excluded from the study if they exhibited (1) cerebellar lesion, (2) multiple strokes, (3) severe cognitive and perceptual deficit, and (4) any acute medical illness.

This study was a prospective, cross-sectional, and nonexperimental design. The subjects were conveniently selected as per the inclusion criteria. A detailed clinical evaluation was performed and then the standardized assessments were applied. The assessments were carried out by two assessors who had 15 to 20 years of experience in stroke rehabilitation. One of the assessors applied the motor measures for all the subjects, while the other conducted the balance measures for them. Two sessions were allotted for the entire assessments, one for the motor level and the other for the balance assessment. All the assessment procedures were performed as per the standard guidelines of the respective scale.

### 2.2. Outcome Measures

Motor level was assessed by using Brunnstrom recovery stages (BRS) and Fugl-Meyer Assessment (FMA) for the upper and lower extremities [25, 26]. BRS is classified under six categories (1, flaccidity with no movement, to 6, individual joint movement with little awkwardness) as per the motor recovery process of poststroke hemiparesis. The stages have been separately described for the upper extremity (BRS-UE), hand (BRS-H), and lower extremity (BRS-LE). BRS demonstrated strong responsiveness with the Motricity Index (effect size *d* = 0.97, Wilcoxon *Z* = 5.33, and *P* < 0.001; *d* = 0.81, *Z* = 5.09, and *P* < 0.001) [27]. BRS was found to be highly valid (*r* = −0.81; *P* < 0.001) when compared with neurophysiological measures [28]. However, there is no reporting of its reliability in the literature. FMA, a 3-point ordinal scale, measures the impairment of volitional movement ranging from 0 (items cannot be performed) to two (items can be fully performed). The upper extremity section of this scale (FMA-UE) is divided into two subsections: upper arm (FMA-UA) and wrist hand (FMA-WH). The section comprises nine items (6: upper arm; 3: wrist hand) with a sum score of 66 (36: upper arm; 30: hand). The lower extremity section (FMA-LE) has six items with a maximum score of 34. In both the sections, items are further divided into different subcomponents. FMA demonstrated high reliability (*r* = 0.99) and good validity (*r* = 0.63 to 0.88) [29, 30]. It exhibited good responsiveness for the poststroke motor assessment [31].

The balance of the subjects was assessed by Berg Balance Scale (BBS), Postural Assessment Scale for Stroke Patients (PASS), and Functional Reach Test (FRT). BBS is used to assess static and dynamic balance abilities required for functional tasks. It comprises 14 items, scored on a 5-point ordinal scale (0, poor balance, to 4, good balance) with a maximum score of 56. The items range from unsupported sitting/standing to turn 360°/standing upon one leg. The scale demonstrated excellent interrater as well as test-retest reliability (ICC = 0.91 to 0.99) and internal consistency (Cronbach alpha = 0.92 to 0.98), and its validity ranged from 0.55 to 0.91 (*r*) [29, 32, 33]. PASS assesses postural control on a 4-point ordinal scale (0, cannot perform, to 3, can perform independently) comprising 12 items (PASS-T), 5 for maintaining (PASS-M) and 7 for changing the posture (PASS-C). It showed excellent interrater agreement (ICC = 0.97) and high internal consistency (Cronbach = 0.93). Further, it showed acceptable validity, ranging from 0.73 to 0.89 (*r*) [34]. FRT is a quick and performance-based test to assess dynamic postural control during a functional activity. It is measured as a maximal forward-reaching distance beyond the arm's length while maintaining the standing position. The normal range varies from 10.5 to 16.7 inches depending on the age and gender [33]. The reliability of FRT ranges from 0.92 to 0.98 (ICC), while the validity varies from 0.65 to 0.71 (*r*). In the present study, the measurement was performed on the less-affected upper extremity [29].

### 2.3. Data Analysis

Data analysis was performed using IBM SPSS, version 21.0. The Spearman test (*ρ*) was used to find the relation between the measures of motor level and balance. Relation between the individual items of each motor outcome measure with that of the balance measures was analyzed using the same test. The level of relation corresponding to the correlation coefficient was followed as low (<0.5; *P* < 0.05), moderate (0 .5 to 0.69; *P* < 0.05), and high (0 .7 to 0.89; *P* < 0.05) [35]. Furthermore, subgroup and partial correlation analysis were also conducted. The significance level was set at *P* < 0.05.

## 3. Results

All the enrolled subjects completed the assessment protocol. The mean age of the participants was 48.82 ± 12.04 years. The average poststroke duration was 19.73 ± 12.21 months. Twenty-six (59%) subjects had right side paresis. Three subjects (7%) exhibited the hand dominance for left side.  Table 1 shows the detailed demographic characteristics of the participants. Thirty-two (72.5%) subjects were at stages III to IV of BRS-UE, while 31 (70%) were at stages III to IV of BRS-LE. The mean BBS of the subjects was 42.64 ± 10.35.  Table 2 shows the description of the motor level and balance as assessed by the outcome measures.

**Figure 1:**
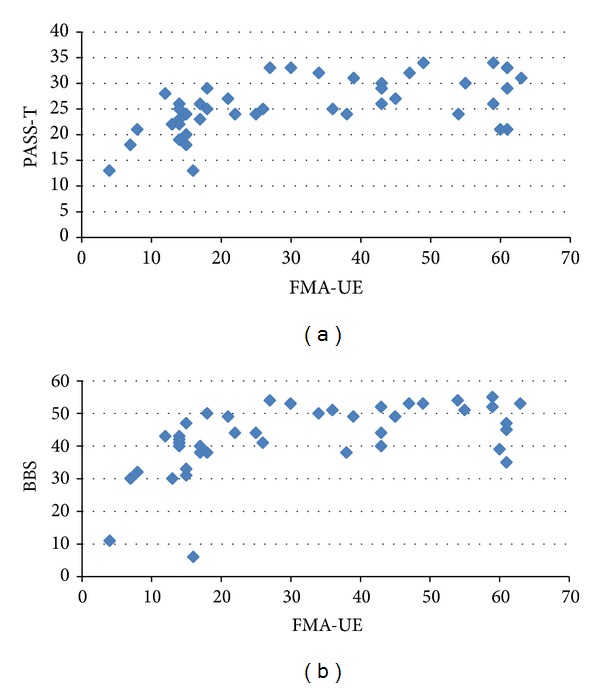
(a) Showing the relation between the total score of Fugl-Meyer Assessment: upper extremity (FMA-UE) and Postural Assessment Scale for Stroke (PASS-T). (b) Showing the relation between the total score of Fugl-Meyer Assessment: upper extremity (FMA-UE) and total score of Berg Balance Scale (BBS).

**Figure 2:**
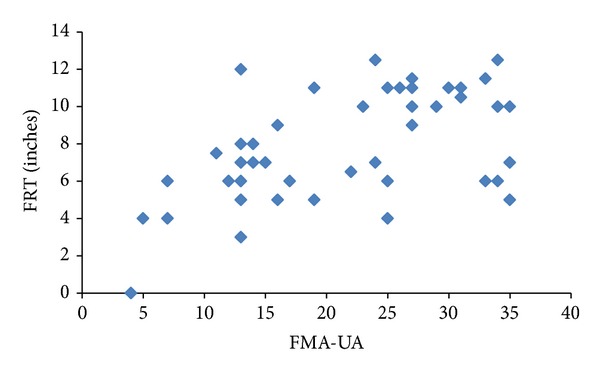
Showing the relation between the upper arm subscore of Fugl-Meyer Assessment (FMA-UA) and Functional Reach Test (FRT).

On analyzing the relation between the measures of motor level and balance, moderate correlation (*ρ* = 0.5 to 0.7) was found between most of the variables. BRS-UE, BRS-H, and BRS-LE showed moderate correlation with BBS (*ρ* = 0.54 to 0.60; *P* < 0.001), PASS (*ρ* = 0.48 to 0.64; *P* ≤ 0.001), and FRT (*ρ* = 0.48 to 0.59; *P* ≤ 0.001). FMA-UE, including FMA-UA, also exhibited moderate correlation with BBS (*ρ* = 0.59 to 0.63; *P* < 0.001) and PASS (*ρ* = 0.54 to 0.62; *P* < 0.001). Figures 1(a) and 1(b) show the relation between the FMA-UE and BBS and PASS-T. FMA-UE along with FMA-WH demonstrated low relation with FRT. However, FMA-UA alone was found to be related to FRT (*ρ* = 0.50; *P* = 0.002).  Figure 2 showed the relation between FMA-UA and FRT. FMA-LE demonstrated fair correlation with BBS (*ρ* = 0.50; *P* = 0.001) and PASS (*ρ* = 0.50; *P* = 0.001). It showed poor relation with PASS-C while showing no relation with FRT.  Table 3 shows the detailed *ρ* value for all the variables.

Further, on exploring the relation between the individual items of FMA with the same of BBS, FMA-UE items II (flexor synergy), III (extensor synergy), and IV (movement out of synergy) were found to have moderate correlation (0.50 to 0.64; *P* = 0.001 to 0.003) with most of the BBS items (except items 1, 2, 3, 5, and 6). FMA wrist-hand items (VII and VIII) also exhibited moderate relation with BBS items 4, 7, and 9 (0.50 to 0.58 and *P* < 0.001 to 0.002). FMA IX showed moderate relation with BBS items 4 to 12 (ranging from 0.50 to 0.65; *P* < 0.001 to 0.002). FMA-UE items II, III, IV, and IX demonstrated significant correlation with total BBS (0.46 to 0.66; *P* < 0.001 to 0.002). All items of FMA-UE (except I and II) showed good relation with PASS item 3 (standing with support) (0.50 to 0.58; *P* < 0.001). FMA-UE items II to V were found to be significantly related (0.45 to 0.66; *P* < 0.001 to 0.003) with PASS-C items 6 to 10. FMA-UE II to IV also exhibited moderate correlation with total PASS score (0.55 to 0.65; *P* < 0.001). FMA-UE VII, VIII, and IX demonstrated correlation with PASS item 6 (0.45 to 0.53 and *P* < 0.001 to 0.002). FMA-UE VII and IX were found to be related to PASS item 10 (0.45 to 0.47 and *P* < 0.001 to 0.002). Same components of FMA-UE showed good relation with total PASS (0.50 to 0.51; *P* < 0.001). FMA-UE items II, III, and IV displayed low relation with FRT (0.45 to 0.48; *P* < 0.001 to 0.002). All other FMA-UE items did not demonstrate any relation with FRT.

**Figure 3:**
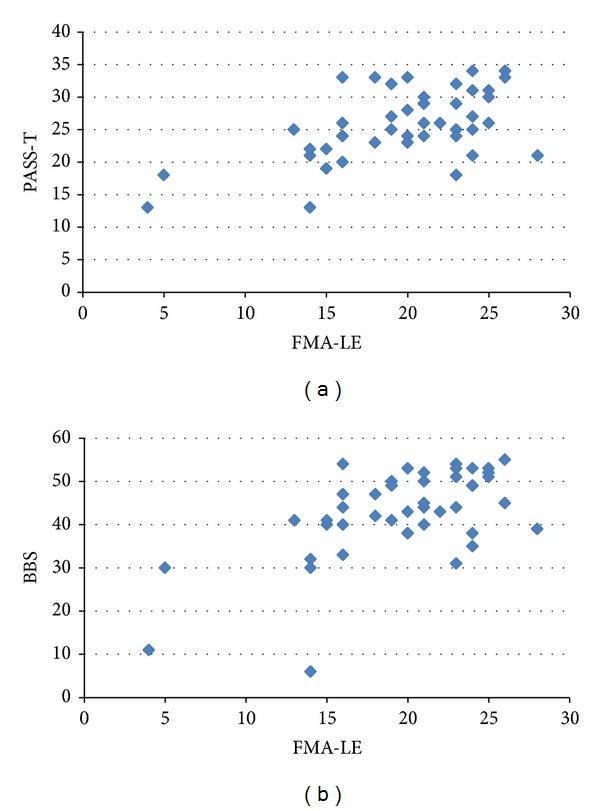
(a) Showing the relation between the total score of Fugl-Meyer Assessment: lower extremity (FMA-LE) and Postural Assessment Scale for Stroke (PASS-T). (b) Showing the relation between the total score of Fugl-Meyer Assessment: lower extremity (FMA-LE) and total scores of Berg Balance Scale (BBS).

BBS and FMA-LE item II (flexor synergy) were found to be related to BBS items 5, 8, 11, 12, and 14 and overall total BBS (*ρ* = 0.50 to 0.60 and *P* < 0.001 for all). FMA-LE item IV showed significant correlation with two items of BBS (8 and 14) and total BBS (*ρ* = 0.45 to 0.47 and *P* = 0.001 to 0.002). Only 2 FMA-LE items (III and IV) were found to be related to PASS items 3, 4, 8, 9, and 10 (*ρ* = 0.47 to 0.58 and *P* ≤ 0.001). The items also related to PASS-M (*ρ* = 0.49 to 0.53; *P* ≤ 0.001) and total PASS score (*ρ* = 0.48 to 0.52 and *P* ≤ 0.001). Figures 3(a) and 3(b) show the relation between the FMA-LE and BBS and PASS-T. None of the items of FMA-LE was found to be related to FRT.

The scores of balance measures were not found to be significantly different between the subgroups based on the side of involvement (dominant/nondominant side) and gender. Only FRT of male subjects (8.44 ± 2.98 inches) was found to be higher than the female subjects (5.95 ± 1.80 inches) with *P* < 0.008.

Partial correlation analysis was also performed to neutralize the effect of FMA-UE and FMA-LE on one another, when inferring the relation with balance measures. By controlling FMA-LE, no relation was found between FMA-UE and BBS, while, on controlling FMA-UE, FMA-LE demonstrated low significant relation with BBS (*ρ* = 0.38; *P* < 0.012). Both FMA-UE and FMA-LE exhibited no relation with PASS-T when one of the FMA (FMA-LE / FMA-UE) components was controlled.

## 4. Discussion

Balance is a complex motor behavior and involves multiple sensorimotor, environmental, and functional contexts, required during functional performance [9]. In other words, all factors, such as biomechanical constraint, cognition, perception, somatosensation, and motor control, affect the balance ability. Apart from the paretic lower limb, the upper extremity may also be responsible for poor balance [13, 15].

To date, no study has inferred the relation between the motor level of the limbs and balance in stroke. Most of the studies have been done focusing either on the weight-bearing and functional issues or on the validity aspect of a measure [11, 12, 14, 36, 37]. This study utilized multiple measures for assessing motor level and balance. The three balance measures were used to examine the various simple to complex balance-related tasks. In addition, the relation was investigated between individual movement and balance components. The findings of the present study revealed a positive relation between the motor level of the paretic limbs and balance in stroke subjects.

After stroke, the paretic upper limb is unable to execute voluntary movements, usually needed before and during losing the balance. The findings of the present study indicated that the lower the motor level of the upper limb the poorer the balance. Although the role of arm movement in balance control is evident to the healthy individuals, no study confirms the same in stroke subjects [15, 16].

Due to hemiparesis of the body, the centre of gravity shifts to the stronger side of the body. Weight-bearing asymmetry is the most common reason proposed for balance deficit among stroke survivors [11, 12]. The lower extremity recovery level exhibited moderate relation with BBS and PASS-T. However, FMA-LE assesses only voluntary movements of the lower limb and does not have any weight-bearing item. It may be inferred that the ability to carry out voluntary leg movements is necessary for maintaining balance, for example, the stepping strategy, the ability to take forward or sideward step to prevent falling [33].

Fall is the most common complication in poststroke subjects, and the fear of fall or anticipatory behaviour leads to reduced physical activity [38]. The average BBS of the study participants was 42.6. The scores below 45 were considered as increased risk for falls [39]. However, the relation of BBS scores between fallers and nonfallers is still controversial [20]. In the present study, 18 (41%) subjects reported fear of fall, 14 (32%) had history of occasional fall, and 12 (27%) had the frequent fall prior to the rehabilitation management. All the participants were undergoing conventional rehabilitation for more than 3 months. None of the subjects reported fall after the commencement of the management.

FRT was not found to be related to both FMA-UE and FMA-LE. This could be due to the assessment of FRT using the less-affected upper limb. In comparison to other balance measures, FRT assesses only reaching ability. BBS, apart from reaching, comprises multiple balance-related tasks in static and dynamic positions. PASS exclusively measures stroke-related postural impairment. Although some of the items overlap with that of the BBS, PASS specifically assesses paretic side and bed mobility.

Simultaneous recovery of the affected upper and lower limbs may influence the balance control. In the present study, the effect of motor level of one limb (upper or lower) was controlled while analyzing the relation of recovery with balance. It could be inferred that the balance is not related to either upper or lower limb independently. Rather, balance is an integrated response of the performance of the upper extremities while maintaining upright position by the lower extremities.

A good correlation was found between the recovery of affected limb (upper limb and lower limb independently) and balance. However, the level of relation declined when both the affected limbs were considered as a single unit. Both FMA-UE and FMA-LE exhibited no relation with PASS-T when one of the FMA (FMA-LE/FMA-UE) components was controlled.

FMA-UE components exhibited good relation with the majority of BBS items. The items were either those which required control through protective extension such as stand-to-sit or complex items (9 and beyond) comprising the upper limb control and manipulation. Similarly, FMA-UA components exhibited good relation with most of the PASS-C items, which required the upper extremity control during postural change. Both synergistic and nonsynergistic FMA-LE components demonstrated acceptable association with BBS items such as reaching forward while standing and standing upon one leg.

Different approaches such as task-oriented gait training are used to alleviate balance dysfunction in stroke [40]. Despite evidence, no single approach is considered to be the best to achieve balance in stroke subjects [41]. The arm training improves postural control and independent walking [42, 43]. Exclusive task-oriented arm training in standing may improve postural control (centre of pressure displacement and the anticipatory adjustments) [42, 44]. The role of the limbs in balance control may be utilized in stroke rehabilitation. The present study had few limitations, for instance, variability in age, chronicity, and lesioned area. The number of female participants was considerably low. The trunk impairment may also affect balance. However, the trunk control was not assessed in the study. Further, due to nonavailability of the reliability, only the validity as a psychometric value was considered for BRS.

Advance measures such as kinematic analysis and force place may be used for future studies. Interaction of rehabilitation intervention on recovery and balance may be investigated in the longitudinal studies.

## 5. Conclusion

Balance, a multifactorial phenomenon, required for safe functional performance, gets impaired in stroke. In the present study, there exists a positive relation between the motor level of the affected upper and lower extremities and balance among poststroke subjects. Voluntary motor control of the paretic limbs may be one of the factors in balance-related functions. The findings may be utilized in planning stroke rehabilitation program.

## Figures and Tables

**Table 1:** Demographic characteristics of the participants.

Characteristics	Number (%)/mean ± SD
Number of participants (44)	
Male/female	34 (77%)/10 (23%)
Age (in years)	48.82 ± 12.04
Poststroke duration (in months)	19.73 ± 12.21
Side of involvement (right/left)	26 (59%)/18 (41%)
Handedness (right/left)	41 (93%)/03 (07%)

Type of stroke	
Ischemic/hemorrhagic	28 (64%)/16 (36%)

Area of involvement	
Frontoparietal	12 (27%)
Basal ganglia	04 (09%)
Thalamus	06 (14%)
Internal capsule	02 (4.5%)
Multiple	20 (45.5%)

Risk factor	
Hypertensive	32 (77%)
Hereditary	15 (34%)
Smoking	12 (27%)
Alcoholic	17 (39%)
Diabetic	08 (18%)
Obesity	07 (16%)

SD: standard deviation.

**Table 2:** Description of motor recovery and balance measures.

Outcome measures	Stage I	Stage II	Stage III	Stage IV	Stage V	Stage VI
BRS-A	00 (0%)	04 (9%)	20 (45.5%)	12 (27%)	07 (16%)	01 (2%)
BRS-H	00 (0%)	20 (45.5%)	10 (23%)	07 (16%)	06 (13.5%)	01 (2%)
BRS-LE	01 (2%)	02 (4.5%)	12 (27%)	19 (43%)	10 (23%)	00 (0%)

	Mean ± SD					

FMA-UE (*maximum score*—*66*)	31.98 ± 18.92					
FMA-UA (*maximum score*—*36*)	21.59 ± 9.23					
FMA-WH (*maximum score*—*30*)	10.39 ± 9.51					
FMA-LE (*maximum score*—*34*)	19.64 ± 5.10					
BBS (*maximum score*—*56*)	42.64 ± 10.35					
PASS-T (*maximum score*—*36*)	25.75 ± 5.30					
PASS-M (*maximum score*—*15*)	10.45 ± 2.18					
PASS-C *(maximum score*—*21) *	15.30 ± 3.46					
FRT (*in inches*)	7.87 ± 2.94					

BRS: Brunnstrom recovery stages, A: arm, H: hand, FMA: Fugl-Meyer Assessment, UE: upper extremity, UA: upper arm, WH: wrist and hand, BBS: Bergs Balance Scale, PASS: Postural Assessment Scale for Stroke Patients, T: total, M: maintenance of posture, C: change in posture, FRT: Functional Reach Test, and SD: standard deviation.

**Table 3:** Relation between the motor recovery and balance measures.

	BBS	PASS-C	PASS-M	PASS-T	FRT
BRS-A	*ρ* = 0.60 (*P* < 0.001)	*ρ* = 0.64 (*P* < 0.001)	*ρ* = 0.55 (*P* < 0.001)	*ρ* = 0.63 (*P* < 0.001)	*ρ* = 0.59 (*P* < 0.001)
BRS-H	*ρ* = 0.55 (*P* < 0.001)	*ρ* = 0.50 (*P* = 0.001)	*ρ* = 0.50 (*P* = 0.001)	*ρ* = 0.52 (*P* < 0.001)	*ρ* = 0.55 (*P* < 0.001)
BRS-LE	*ρ* = 0.54 (*P* < 0.001)	*ρ* = 0.51 (*P* < 0.001)	*ρ* = 0.55 (*P* < 0.001)	*ρ* = 0.57 (*P* < 0.001)	*ρ* = 0.48 (*P* = 0.001)
FMA-UE	*ρ* = 0.59 (*P* < 0.001)	*ρ* = 0.56 (*P* < 0.001)	*ρ* = 0.54 (*P* < 0.001)	*ρ* = 0.60 (*P* < 0.001)	*ρ* = 0.38 (*P* = 0.01)
FMA-UA	*ρ* = 0.63 (*P* < 0.001)	*ρ* = 0.59 (*P* < 0.001)	*ρ* = 0.58 (*P* < 0.001)	*ρ* = 0.62 (*P* < 0.001)	*ρ* = 0.50 (*P* = 0.002)
FMA-WH	*ρ* = 0.50 *P* = 0.001	*ρ* = 0.50 *P* = 0.001	*ρ* = 0.43 *P* < 0.003	*ρ* = 0.50 (*P* < 0.001)	*ρ* = 0.30 *P* < 0.04
FMA-LE	*ρ* = 0.50 (*P* = 0.001)	*ρ* = 0.41 (*P* = 0.006)	*ρ* = 0.50 (*P* = 0.001)	*ρ* = 0.50 (*P* = 0.001)	*NS *

BRS: Brunnstrom recovery stages, A: arm, H: hand, FMA: Fugl-Meyer Assessment, UA: upper arm, WH: wrist and hand, UE: upper extremity, PASS: Postural Assessment Scale for Stroke Patients, C: change in posture, M: maintenance of posture, FRT: Functional Reach Test, BBS: Berg Balance Scale, *ρ*: Spearman test, and NS: not significant.
